# Surgical, oncologic, and obstetric outcomes of radical trachelectomy in early-stage cervical cancer: results from a retrospective cohort study at Brazil National Cancer Institute

**DOI:** 10.3389/fonc.2024.1267625

**Published:** 2024-03-08

**Authors:** José Augusto Bellotti, Isabella Gonçalves Gutierres, Yara Lúcia Furtado, Patricia Patury, Juliana de Almeida Figueiredo, Gustavo Guitmann, Rossano Keppler Alvin Fiorelli, Fernanda Campos da Silva

**Affiliations:** ^1^ Department of Gynecologic Oncology, National Cancer Institute, Rio de Janeiro, RJ, Brazil; ^2^ Department of Gynecology and Obstetrics, Rio de Janeiro State Federal University, Rio de Janeiro, RJ, Brazil; ^3^ Department of Internal Medicine, University of Brasília, Brasília, DF, Brazil; ^4^ Department of Gynecology, Federal University of Rio de Janeiro, Rio de Janeiro, RJ, Brazil

**Keywords:** cervical cancer, trachelectomy, fertility preservation, gynecologic surgical oncology, pregnancy

## Abstract

**Objective:**

to analyze oncological, obstetrical, and surgical results of young early-stage cervical cancer patients who underwent radical trachelectomy (RT) surgery and wished to maintain their fertility.

**Methodology:**

a retrospective cohort study was carried out concerning cases attended at the Brazilian National Cancer Institute Gynecology Oncology Service. Patients who underwent RT between January 2005 and January 2021 were included.

**Results:**

A total of 32 patients with median age of 32 years old, 62.5% of whom were nulliparous, were assessed. Concerning cancer type, 65.6% squamous cell carcinoma (SCC) cases, 31.2% adenocarcinoma cases and 3.1% adenosquamous carcinoma cases were verified. Stage IA2 was evidenced in 12.5% of the patients and stage IB < 4 cm in 87.5%. Regarding surgical approaches, 68.25% of the patients underwent vaginal RT (VRT), 18.75%, abdominal RT (ART), 9.3%, the robotic radical trachelectomy (RORT) and 3.1%, video laparoscopy radical trachelectomy (VLRT). The median number of removed lymph nodes was 14, with only two detected as positive. Two cases of positive surgical margins were noted. A total of 3.1% intraoperative and 31.25% postoperative complications were observed, with cervical stenosis being the most common. The recurrence rate of the study was 3.1%, with a median follow-up time of 87 months, where 3.1% deaths occurred. The pregnancy rate of the study was 17.85% (5/28), with 54.5% evolving to live births and 45.5% evolving to abortion.

**Conclusion:**

Radical trachelectomy is a feasible procedure presenting good oncological results and acceptable pregnancy rates.

## 1 Introduction

The diagnosis of early cervical cancer is still a major challenge. This type of cancer ranks fourth among the most common cancers and is the fourth cause of death from cancer in women worldwide (1). In Brazil, this type of neoplasm ranks third among women, excluding cases of non-melanoma skin cancer. A total of 16,710 new cases were estimated to be diagnosed in 2022 (2).

In the United States, about 38.3% of new cervical cancer cases are diagnosed in women younger than 45 every year (3). Many of these young patients are nulliparous or have incomplete families and wish to preserve their fertility. The management of this type of patient is a challenge for oncological gynecologists and should always be approached in a multidisciplinary manner. Surgeries in this regard comprise a therapeutic option for fertility preservation, including radical trachelectomy (RT) and conization/simple trachelectomy and, more recently, neoadjuvant chemotherapy followed by cervical resection (4). Embryo and unfertilized oocyte cryopreservation can also be carried out (5). The indicated treatment varies according to disease staging, following International Federation of Gynecology and Obstetrics (FIGO) guidelines (6).

In this scenario, RT has been gaining ground as a safe alternative for cervical cancer treatment in women who wish to maintain their reproductive potential, with similar oncological results to hysterectomies (7, 8). Therefore, RT, regardless of the access route, is currently considered the surgical treatment of choice for young early-stage cervical tumor patients who wish to preserve their fertility (9).

Radical trachelectomy surgery involves the removal of cervix, upper third of the vagina, and parametrium lymph nodes. The uterine body is preserved and the proximal vaginal stump is then sutured to the uterine body (10). The performance of the cerclage is recommended. The term “radical” refers to parametrial resection. The amount of resected parametrial tissue is still a matter of controversy and a trend towards smaller resections is noted (11).

The first RT was performed in 1987 by Daniel Dargent, through the vagina employing video laparoscopic lymphadenectomy (12). From this point onwards, trachelectomies began being performed through the abdominal (1993), total laparoscopic (2003) and robotic (2008) routes (13).

The criteria for RT are the presence histological type squamous cell carcinoma (SCC), adenocarcinoma or adenosquamous carcinoma, the desire to preserve the ability to conceive, age below 40, disease staging up to stage IA1 with lymphovascular invasion, IA2 or IB < 4 cm, negative lymph nodes and tumors preferably smaller than 2 cm (14).

A low recurrence rate of about 3% is expected following RT (15, 16), with an estimated pregnancy rate of about 20%, according to Smith et al. in a systematic review (15). Theses pregnancies are, however, associated with an increased risk of obstetric complications, including preterm delivery, premature membrane rupture and abnormal varicose vein bleeding at the uterovaginal anastomosis site (17).

In this context, the initial hypothesis of this study is that RT presents similar oncological results to standard surgery, resulting in reasonable obstetric result.

The general objective of this study was to analyze oncological (overall survival and disease-free survival) and obstetric (pregnancy rate and live birth rate) outcomes in women undergoing surgical cervical cancer treatment. Secondary objectives included the analysis of factors associated to the surgical technique, such as surgical time, intra- and postoperative complications, length of hospital stay, number of removed lymph nodes and surgical margins.

## 2 Methodology

A retrospective cohort study was carried out concerning women undergoing surgical cervical cancer treatment at the Brazilian National Cancer Institute’s (Instituto Nacional de Câncer, INCA) Gynecology Oncology Service from January 2005 to January 2021.

Patient diagnosis was based on uterine cervix biopsies or on conization specimens, and all cases were reviewed by the INCA’s pathological anatomy service. The INCA’s Gynecology Oncology Service Radical Trachelectomy criteria are depicted in **Table 1**.

**Table 1:** Brazilian National Cancer Institute Gynecology Oncology Service Radical Trachelectomy eligibility criteria.

Item	Criterion
1	Desire to preserve fertility
2	Histological confirmation of an invasive tumor
3	Histological types: squamous cell carcinoma, adenocarcinoma, adenosquamous carcinoma
4	Staging according to FIGO 2009: IA1 (with lympho-vascular involvement), IA2 or IB < 4 cm
5	Tumor smaller than 20 mm and located on the outer surface of the cervix
6	Absence of lymph node or distant metastasis

Data were obtained from INCA’s electronic and physical medical records. The assessed variables were age, pregnancies, births and previous abortion, histological tumor type, tumor grade. FIGO stage system used was 2009 version.

because most of the patients were include before 2018 (84%). Surgical/oncological variables comprised surgery date, type of surgical approach, size of the residual tumor, number of removed lymph nodes, number of positive lymph nodes, surgical limits, the presence of lymphovascular invasion, intra- and postoperative complications, length of the patient’s hospital stay, the need for adjuvant treatment, recurrence, and date of last consultation. Obstetric data on pregnancies, abortions and live births after surgery were also analyzed.

This project was approved by the Ethics and Research Committee and included in the Brasil Plataform under no. 49953415.1.0000.5258.

### 2.1 Data analysis

A descriptive data assessment was carried out using central tendency measures for continuous variables and relative and absolute frequencies for categorical variables. Surgical time was presented as mean and standard deviation (SD). Other measures were demonstrated as median and range.

The R v. 3.4.4 and Jamovi v. 2.2.5 softwares were used for all statistical analyses. Fisher’s exact test was applied for a comparative analysis between surgical techniques and categorical variables (*i.e*., pregnancy, complications, and recurrence). The Shapiro-Wilk test was applied to assess the distribution of continuous variables, such as duration of surgery and length of hospital stay. When normally distributed, the ANOVA test was used to compare variances between group means for each surgical approach. The Kaplan-Meier analysis could not be performed to assess survival, due to the small sample size. p values < 0.05 were considered statistically significant in all analyses.

## 3 Results

A total of 32 patients who underwent RT were identified at INCA between January 2005 and January 2021. Patient and case characteristics are listed in **Table 2**.

**Table 2:** Cancer patient and case characteristics.

Characteristics	
Age (years)	
Means	31 (19–44)
Nulliparous	20 (62.5%)
Tumor staging	
IA2	4 (12.5%)
IB < 4 cm	28 (87.5%)
Histological type	
Squamous cell carcinoma	21 (65.6%)
Adenocarcinoma	10 (31.2%)
Adenosquamous carcinoma	1 (3/1%)

### 3.1 Surgical results

The median number of removed lymph nodes was 14. No compromised lymph nodes were identified in frozen sections during surgery, as per INCA’s institutional routine. Although two cases (6.2%) presented positive lymph nodes in the final anatomopathological exam (paraffin), with indication of adjuvant radiotherapy, chemotherapy and brachytherapy. Lymphovascular invasion was identified in only three (9.3%) cases (**Table 3**).

**Table 3:** Relation of surgical findings and intra and postoperative complications.

Result	Number
Removed lymph nodes	
Median	15 (5-30)
Positive	2 cases
Residual tumor size	
Median	11 (2-25)
≤ 20 mm	19 (59.4%)
> 20 mm	4 (12.5%)
Not identified	9 (28.3%)
Lymphovascular invasion	
Present	3 (9.3%)
Absent	29
Surgical margins	
Compromised	2 (6.2%)
Non-compromised	30
Intraoperative complications	1 (3.1%)
Post-surgical complications	
Cervical canal stenosis	7 (21.9%)
Ureterovaginal fistula	1 (3.1%)
Vesico-vaginal fistula	1 (3.1%)
Lower limb lymphedema	2 (6.2%)
Chronic pain	1 (3.1%)
Lymphocele	1 (3.1%)

A 3.1% (1/32) intraoperative complication rate and 40.62% (13/32) postoperative complication rate were noted, albeit with no statistical significance by Fisher’s exact test when analyzing surgical approach effects (p=0.307).

Regarding intraoperative complications, a bladder injury was noted in one case, identified, and treated during surgery. The most frequent postoperative complication was cervical canal stenosis, observed in seven (21.88%) cases, followed by fistulas in two (6.25%) cases, comprising a ureterovaginal fistula and a vesicovaginal fistula. Furthermore, two persistent lower-limb lymphedema cases, one chronic pelvic pain case and one lymphocele case were observed, the latter two the same patient (**Table 3**).

No compromised surgical margins were identified in frozen section samples during surgery. Subsequent anatomopathological analyses however, identified compromised surgical margins in two (6.2%) cases, with hysterectomy complementation indicated.

Residual tumors in surgical specimens were identified in 24 cases (75%), with six (25%) presenting *in situ* neoplasia.

The following surgical approaches were employed: radical trachelectomy with pelvic lymphadenectomy via abdominal approach (ART), vaginal approach with videolaparoscopic pelvic lymphadenectomy (VRT), robotic approach (TRRO) and radical trachelectomy and pelvic lymphadenectomy totally via videolaparoscopic approach (VLRT) (**Table 4**).

**Table 4:** Radical Trachelectomy Route, number of cases (N) and surgical time.

Surgery type	N	Mean time (SD)
Total	32 (100%)	248 (59)
Abdominal trachelectomy (ART)	6 (18.75%)	278 (68,09)
Robotic trachelectomy (RORT)	3 (9.38%)	260 (38,83)
Laparoscopic radical trachelectomy (VLRT)	1 (3.13%)	300 (-)
Vaginal trachelectomy and laparoscopic pelvic lymphadenectomy	22 (68.75%)	255 (57,16)

The mean surgical time was 248 minutes, detailed for each surgical route in **Table 4**. The surgical time data distribution was normal (P=0.154 by the Shapiro-Wilk test), so an ANOVA test was applied, indicating a non-statistically significant (p=0.286) difference between the mean times for the different surgical approaches. This indicates that the type of surgery (independent variable) does not significantly influence surgical time (dependent variable).

The median hospital stay for surgery was 4 days and the median post-surgery hospital stay was 3 days. The median hospital stays for each approach were as follows: for ART, 7 days, for VRT, 5 days, TRRO, 4 days and for the single VLRT case, 5 days. The hospitalization distribution data was non-normally distributed (P=0.05 by the Shapiro-Wilk normality test), so these values could not be compared.

The median of follow-up time was 87 months, with data collected up to May 2022. Three patients (9.3%) were lost during follow-up. Concerning the remaining 29 (90.62%) cases, one (3.4%) death and three (10.3%) completions with total hysterectomy were noted, as well as 10 (34.48%) institutional discharges after 5 years without evidence of recurrence, while 15 (51.72%) cases are still in follow-up (**Table 5**).

**Table 5:** Relation of oncological outcomes.

Oncological outcomes	N = 29
Recurrence	1 (3.4%)
Deaths	1 (3.4%)
Adjuvant treatment	2 (6.9%)

### 3.2 Oncological results

Only one recurrence and death case were observed after surgery in the present study. This case evolved with local recurrence and nodal and lung metastasis about 84 months after surgery. This case, without any adverse prognostic factors, was a grade II squamous cell carcinoma (CEC), with clear margins, 10 negative excised lymph nodes and no lymphovascular invasion. The patient was treated with six chemotherapy sessions with the administration of Carboplatin and Taxol, evolving to death 6 months after the beginning of palliative treatment.

### 3.3 Obstetrics results

Of the 32 patients, four were excluded from the obstetric results, two due to hysterectomies and two for having undergone radiotherapy, brachytherapy, and adjuvant chemotherapy due to positive lymph nodes. A final fertility rate of 17.85% was observed, with five pregnant patients, two of whom were pregnant more than once, totaling nine pregnancies. Of these, four (45.5%) evolved to abortion and five (54.5%), to live birth. Of the five patients who became pregnant, three underwent VRT and two, ART. The pregnancy rate following VRT was 15.7% (three of 19 patients able to conceive) and following ART, 33.33% (three of six patients able to conceive), with zero for the other routes (P= 0.763, with a 95% confidence interval by Fisher’s exact test). **Table 6** depicts the obstetric outcome data.

**Table 6:** Relation of obstetric outcomes.

Number of patients included	32
Number of patients with fertility sparing who remained under follow-up	28 (87.5%)
Number of patients who became pregnant	5 (17.85%)
Obstetric outcomes	
Pregnancy rate	N = 9
Abortion	4/9 (45.5%)
Live birth	5/9 (54.5%)

## 4 Discussion

Fertility preservation in young early-stage cervical cancer patients is a challenge. Our study suggests that RT is an adequate approach from an oncological and obstetric point of view, corroborating American (18), European (8) and in Brazilian Federation of Gynecology and Obstetrics (FEBRASGO) (19) guidelines as a therapeutic possibility for this type of patient.

Younger early-stage cervical cancer patients that wish to preserve their fertility have been reported previously in the literature. For example, Smith et al. in a 2020 systematic review, reported a median age of 31 for these patients (15), as did Kiss et al., Guo et al., Wethington et al. (20–22), similar to the age as reported herein. The fact that most patients are nulliparous seems to be common and is related to the desire to maintain pregnancy potential. Smith et al. and Kiss et al. reported 64.1%, 66.67% of nulliparous cases, respectively, similar to the 62.5% frequency reported herein (15, 20).

Regarding the FIGO 2009 staging classification, most cases in the present study were categorized as FIGO stage IB1 (87.5%), in line with Wethington et al., who reported 87% of patients in this stage (22), and Yoshino et al., who indicated 83.3% in their ART assessment (23). Bentivegna et al. reported 78% and 85% of VRT and ART patients in stage IB1 (24) in a 2016 systematic review, while Smith et al, including VRT, ART and laparoscopic RT, reported 74.8% of patients in stage IB1 in a 2020 systematic review (15).

Historically, more SCC cases are noted compared to other histological cervical cancer types, as was the case herein. Smith et al. indicated that most cases were diagnosed as SCC, at 68.5% (15), similar to Yoshino et al. in a retrospective cohort study, at 88.1% (23), the former similar to the SCC frequency of the present study, of 65.6%. Despite this, an apparent upward trend concerning adenocarcinoma cases is noted (25, 26), possibly due to the fact that cytology screenings are more effective in tracking precursor SCC lesions compared to adenocarcinoma (26), and the fact that cytopathologists are now more experienced in detecting adenocarcinomas, which in the past were mistakenly diagnosed as SCC (27).

### 4.1 Surgical outcomes

The presence of lymphovascular invasion, despite not being a contraindication to RT, is considered a risk factor for nodal involvement and, therefore, a reserved prognostic factor (28–30). Prevalence rates ranged from 8.2% to 55% in studies concerning the presence of lymphovascular invasion in trachelectomy specimens (20, 23, 31). In a meta-analysis, Feng et al. reported a 40.4% invasion rate (16), while a 6.6% lymphovascular space invasion rate was noted herein.

Small tumors are often excised completely during diagnostic conization. Thus, it is not uncommon for no tumor to be observed in surgical specimens following RT. The absence of residual tumors, similar to radical hysterectomy cases, seems to be associated with better prognoses (32). Plante et al., in a study of 72 VRT cases, indicated a 60% frequency of cases with no residual disease observed in surgical specimens (33). In the present study, eight (25%) cases with no residual surgical specimen tumors were detected. Among the patients that did present residual tumors in surgical specimens, the lesions ranged from 2 to 25 mm, averaging 14 mm. Feng et al., reported that 82.9% of all analyzed tumors in a meta-analysis were smaller than 2 cm (16).

Assessing nodal status and surgical margins during surgery is paramount towards preserving fertility. The presence of disease-affected pelvic lymph nodes identified during surgery is a contraindication to RT (6). In this regard, Plante et al. reported 10% nodal metastasis cases, with 36% of these identified only in paraffin, with subsequent radiochemotherapy indication (33). Feng et al. reported 5.1% of positive lymph nodes following RT in a meta-analysis (16), while Kiss et al. indicated a median of 38 removed lymph nodes, ranging from 13 to 58 (20) an Malmstem et al, when assessing 28 V RT cases, indicated a mean of 22 resected lymph nodes (34). In the present study, a median of 14 resected pelvic lymph nodes was noted, with a minimum of five and a maximum of 30. Two (6.2%) nodal metastasis cases were identified in the final anatomopathological examination. Adjuvant therapy with radiotherapy, chemotherapy and brachytherapy were then indicated.

When the surgical specimen displays compromised margins following the final anatomopathological analysis, a hysterectomy is indicated. Patients must, therefore, be informed of these possibilities before the surgery, through informed consent forms. Kiss et al. reported one case out of 18 that had to undergo a hysterectomy (20), and Wethington et al. reported 10 patients out of 101 who required hysterectomies due to compromised margins (22). In the present study, two hysterectomy complementation cases were observed, due to compromised endocervical margins.

Regarding route and surgical approaches, RT can be performed vaginally, abdominally, laparoscopically, or robotically. Smith et al. concluded in a 2020 systematic review that, although the literature is limited to case series and reports, oncological outcomes, such as recurrence, recurrence-free survival and overall survival rates, are favorable, with no significant differences observed between surgical approaches (15). In a more recent systematic review conducted in 2022, Morice, Maulard, Scherier et al. reported a similar recurrence rate between the three approaches in FIGO 2018 stage IB1 patients, namely 4.7% vaginally, 2.4% abdominally and 5.2% laparoscopically (4).

Abdominal RT presents several advantages, as it is performed by professionals in units that do not have laparoscopic technology, allows a wider parametrial resection and is the most indicated in cases with tumors between 2 and 4 cm when compared to VRT, while disadvantages include lower fertility rates (16). It is possible that the wider paracervical tissue resection employed in this approach may impair patient fertility.

Minimally invasive approaches, *i.e.*, laparoscopic RT and robotic RT, have the advantages of avoiding abdominal incisions, with less postoperative pain, lower adhesion rates and intraoperative bleeding (15, 35). However, after the Laparoscopic Approach to Cervical Cancer study (LACC *trial*) (36), a prospective multicenter study that compared minimally invasive hysterectomies with abdominal hysterectomies in stage I cervical cancer patients, concerns were raised regarding the possibility of worse oncological outcomes.

Herein, only four minimally invasive surgery cases were observed, three RORT and one VLRT. One (33.33%) of the RORT cases required hysterectomy complementation, due to compromised surgical margins, while the other three cases do not present any sign of recurrence so far. As this is a very small sample size, no literature comparisons were possible.

Some advantages regarding the vaginal route are noted compared to other approaches, such as higher pregnancy rates and obstetric results associated with shorter surgery times (15). As noted in the present study, the vaginal route is generally the most employed for RT (15, 24).

Regarding surgery time, the median surgery time reported by Smith et al. in a systematic review was 226 minutes, with the lowest duration noted for VRT, of 180 minutes. ART was conducted in 240 minutes and VLRT, in 272 minutes (15). These are similar to the median operating time observed in the present study.

Intra- and postoperative RT complication rates are relatively low and consistent with radical hysterectomy results. Feng et al. demonstrated no differences between RT and hysterectomy outcomes, reporting 15% vs 10% of intraoperative complications and 12.3% vs 14.7% of postoperative complications, respectively (16).

Vieira et al. reported three intraoperative complications (3%): one bladder injury and one fallopian tube injury requiring unilateral salpingectomies in the minimally invasive group and one vascular injury in the ART group. These authors also reported postoperative complications such as urinary tract infections (11%), voiding dysfunction (5.4%), lymphocyst formation (4%), cerclage erosion (17.5%) and cervical stenosis (9%). Kiss et al. indicated one intraoperative complication, also a bladder injury, as well as 33% voiding dysfunction, 11% lymphoceles, 5% pelvic peritonitis, 5% wound infection, 5% cervical stenosis, 11% amenorrhea and 5% pelvic abscess. Guo et al. reported 3.5% of intraoperative complications, with one bladder and one intestinal injury and three vascular injuries. Regarding post-ART complications, a 35.7% rate was observed, with 9.8% voiding dysfunction, 3.5% cervical stenosis, 7.7% lower limb edema, 10.5% lymphocyst and 4.2% menstrual abnormalities. In the present study, 3.1% of intraoperative complications and 34.37% postoperative complication rates were noted.

One of the most common complications following RT is cervical canal stenosis (23, 37, 38). In a systematic review on the incidence of cervical stenosis after trachelectomy, Li et al. reported a median incidence of 10.5%, ranging from 0 to 73.3%. The authors also indicated that the occurrence of stenosis varied according to the approach, cerclage and use of anti-stenosis devices. The highest cervical stenosis rate was observed following the ART route, at 11%. An 8.6% incidence was observed in cases where cerclage was performed, versus 3% in cases without cerclage (P>0.005). This incidence decreased to 4.6% when employing an anti-stenosis device versus 12.7% (P<0.001). Herein, seven out of 32 (21.9%) patients presented this complication. All were treated satisfactorily with cervical dilation. As a prevention for this complication, it is part of the INCA’s service routine to introduce an anti-stenosis device immediately after surgery and leave it in place for about seven days.

The median hospital stay length following ART was 7 days, VRT, 5 days, RORT, 4 days and the single VLRT case, 5 days. These findings are in line with another series of ART cases, with a median hospital stay length of 8 days, ranging from 7 to 14 (21), and also with a VRT series, with 3.7 days, ranging from 1 to 9 (33).

### 4.2 Oncological outcomes

Low recurrence rates are reported in the literature. Smith et al. reported rates from 0 to 9.9% in a systematic review (15), while Feng et al. indicated a 4.8% recurrence rate, a 5-year disease-free survival of 94.9% and a 5-year overall survival of 95.7% in a meta-analysis (16). Maulard and Scherier et al. reported recurrence rates of 5.1%, 4%, 7.5% and 5.3% for VRT, ART, VLRT, RORT respectively, in a 2022 meta-analysis (4) and Salvo et al. reported a 4.8% of recurrence following ART and 6.3% following minimally invasive approaches (P=0.40) in a retrospective multicenter study concerning 646 cases, with disease-free survival rates at 4.5 years of 94.3% and 91.5% for ART and minimally invasive approaches, respectively. These authors also reported disease-free survival rates at 4.5 years of 99.2% for ART and 99% for minimally invasive approaches (39). In the present study, only one recurrence case (3.1%) was observed. Survival analyses could not be performed due to the low number of cases. Simultaneous local and distant recurrence (vagina and cervical lymph node) were noted 84 months after surgery. This case was treated with six Carboplatin cycles alongside Taxol, but the patient died after 6 months. The patient was categorized as stage IB < 4 cm, with no lymphovascular invasion, compromised surgical limits or positive lymph nodes in the final anatomopathological examination. A 4 mm residual SCC grade 2 tumor was detected.

### 4.3 Obstetrics outcomes

When it comes to obstetrics, pregnancy rates are reported as highly variable in the literature, probably due to different calculation methods, as this is influenced by the number of women trying to conceive among all patients undergoing conservative fertility treatment, or because of different variability rates between different approaches. In addition, the VRT approach is associated with higher pregnancy rates among the aforementioned routes (15).

Regarding pregnancies after abdominal radical trachelectomy, Kiss et al., Guo et al., Yoshino et al. and Li et al. reported pregnancy rates of 42.8%. 31.7%, 36.8%, 17.4% respectively (20, 21, 23, 37). Wang et al. and Malmsten et al. revealed a rate of 78.9% and 58.6% of pregnancies, respectively, after the vaginal route approach (34, 40), while, Smith, in turn, indicated a median of 23.9% of postoperative pregnancies, with pregnancy rates of 37.8%, 10% and 9.2% following the vaginal, abdominal and laparoscopic routes respectively, in addition to 75.1% live births in a systematic review (15). Zhang et al., on the other hand, in a meta-analysis, reported a 20.5% frequency (16.8%-24.5%) (41).

In the present study, a pregnancy rate of 17.85% was reported, comprising five out of 28 patients. Of the five patients who became pregnant, three (60%) had been submitted to VRT and two (40%) to ART. The pregnancy rate of each route was, thus, 15.7% for VRT (three of 19 patients able to conceive) and 33.33% for ART (two of six patients able to conceive), albeit with no statistical significance. Four patients were excluded, two who underwent hysterectomies and two, adjuvant treatment. Data regarding the real desire for pregnancy after treatment, however, could not be determined. Consequently, fertility rates were calculated for the total number of patients with preserved fertility, which may explain the lower rate observed herein compared to the literature.

Some factors may interfere with a patient’s ability to conceive, such as adhesions following the abdominal approach, fallopian tube abnormalities, greater paracervical tissue resection and cervical canal stenosis (17, 37). Some reports also indicate Asherman’s syndrome and ovarian failure after ART (42). Furthermore, social, family and physical factors, as well as mental health issues, also significantly influence pregnancy rates and may be prevalent in patients undergoing RT (43). As noted previously, a 21.9% cervical stenosis rate was reported herein and, although the patients were satisfactorily treated, this may have interfered with the pregnancy rate outcome.

Moreover, access to infertility and assisted reproduction specialists is scarce in Brazil, especially in the Unified Health System (*Sistema Único de* Saúde, SUS) (44). In this study, as expected in this scenario, all patients who became pregnant became so spontaneously, which may explain the lower pregnancy rate compared to the literature. Furthermore, due to the urgency in beginning cancer treatment, no previous investigation about the reproductive potential of these patients was conducted.

It should also be noted that two of the five patients (40%) evaluated herein with abortion, a higher rate than reported in the literature. Both patients had undergone ART. One of them had three abortions and the other, one, totaling four (44.44% of nine pregnancies). Zhang et al. reported 24.0% (18.8%-29.6%) abortions after RT in a 2017 meta-analysis (41), Li, et al., 36.7% after ART (37), Yoshino et al., 14.2% after ART (23) and Smith et al., found a median rate of 23.9% and 24.3%, 24.4% and 42.9% for the vaginal, abdominal and laparoscopic routes respectively (15).

It is also important to note that deliveries should be performed via cesarean section in these cases, to avoid removing the prophylactic cerclage and circumvent cervical lacerations (17).

Abortions are relatively common, as well as premature labor and premature oval membrane rupture in RT patients (15, 17). An early theory postulated that this was due to a lack of adequate blood flow to the uterus following uterine artery transection. Studies employing 3D computed tomography (45) and magnetic resonance imaging (46) with contrast have demonstrated that artery ligation actually induces new arterial vascularization and does not affect fetal growth or placental function, suggesting preserved fertility. On the other hand, the amount of remaining cervical tissue may influence premature rupture, while the loss of the mucus plug is associated with loss of a protective barrier and a potential increase in pathogens.

Therefore, radical trachelectomy is a feasible option for young early-stage tumor patients who wish to maintain their fertility, with good oncological outcomes comparable to the gold standard treatment and reasonable obstetric results.

## Data availability statement

The raw data supporting the conclusions of this article will be made available by the authors, without undue reservation.

## Ethics statement

The studies involving humans were approved by Comitê de Ética em Pesquisa (CEP/HUGG) do Hospital Universitário Gaffrée e Guinle. The studies were conducted in accordance with the local legislation and institutional requirements. Written informed consent for participation was not required from the participants or the participants’ legal guardians/next of kin because we only use retrospective data, no harm to participants.

## Author contributions

IG: Data curation, Formal analysis, Funding acquisition, Investigation, Methodology, Project administration, Resources, Software, Supervision, Validation, Visualization, Writing – original draft, Writing – review & editing. JB: Conceptualization, Funding acquisition, Methodology, Project administration, Supervision, Writing – original draft. YdM: Funding acquisition, Supervision, Writing – review & editing. PP: Funding acquisition, Supervision, Writing – review & editing. JF: Funding acquisition, Supervision, Writing – review & editing. GG: Funding acquisition, Supervision, Writing – review & editing. RF: Funding acquisition, Supervision, Writing – review & editing. FdS: Funding acquisition, Supervision, Writing – review & editing.
